# Social Media and Digital Inequity: Reducing Health Inequities by Closing the Digital Divide

**DOI:** 10.3390/ijerph21111420

**Published:** 2024-10-26

**Authors:** Zain Jafar, Jonathan D. Quick, Eszter Rimányi, Godfrey Musuka

**Affiliations:** 1 Trinity College of Arts and Sciences, Duke University, Durham, NC 27708, USA; zain.jafar@duke.edu; 2 Duke Global Health Institute, Duke University, Durham, NC 27710, USA; 3 College of Arts and Sciences, University of North Carolina at Chapel Hill, Chapel Hill, NC 27599, USA; eszternr@live.unc.edu; 4 International Initiative for Impact Evaluation (3ie), Harare 0002, Zimbabwe; gmusuka@3ieimpact.org

**Keywords:** social media, public health, market-driven epidemics, health equity, digital equity, digital divide

## Abstract

As its usage has grown, social media has positively and negatively impacted public health. Although social media presents known harms to mental health and spreads misinformation, it also offers rapid dissemination of public health information, expanded access to health resources, and a robust source of health information. However, these benefits are not equitably shared due in part to the “digital divide” of unequal access and use of information and communication technologies. Measurable inequalities in digital access exist among regions, with an eight-fold difference between Central Africa (9.8% social media penetration) and Northern Europe (80.2% social media penetration). Digital inequalities also differ by gender, age, and socioeconomic classes within countries. Increased digital access has been positively associated with improved health information and outcomes related to chronic diseases, infectious disease outbreaks, and reproductive health. Given the expanding role of social media in public health and the emerging evidence on the health benefits of digital access, we argue for reducing digital inequities by (1) creating an enabling government policy and regulatory environment that views digital health access as a social determinant of health; (2) targeting public and private investments to expand digital access for underserved regions and populations; (3) making digital access and use affordable to low-resource populations; and (4) improving digital competency among these groups through digital literacy programs.

## 1. Introduction

Social media has become a prominent feature in the daily lives of billions of people across the globe. At the end of 2021, there were more than 4.26 billion social media users worldwide, with another 1.75 billion users expected by 2027 [1]. Leading social media platforms have billions of active monthly users: Facebook (2.9 billion), YouTube (2.6 billion), WhatsApp (2.0 billion), Instagram (1.5 billion), WeChat/Weixin (1.3 billion), and TikTok (1.0 billion) [2]. Social media use has significantly expanded in low- and middle-income nations in recent years, where regular use has increased from just 34% of people in 2013 to 53% in 2018 [3]. Over the last two years, Africa has experienced a 13% year-on-year growth in the number of people using the internet [4]. As social media’s reach has grown globally, it has interacted profoundly with public health—both positively and negatively.

Social media has caused serious harm to mental health and perpetuated the rampant spread of misinformation. Widespread misinformation has fueled vaccine hesitancy among social media users and led to attacks on physicians and health officials [5]. These adverse effects have been exacerbated by social media’s market-driven epidemic dynamic of denying evident harms, resisting mitigation efforts, and persisting in the use of addictive algorithms that amply these harms [6].

Social media has also proven itself to be an invaluable public health tool, offering the rapid dissemination of public health information, expanded access to health resources, and other benefits for the public, patients, healthcare providers, and policy-makers. And recent work has focused on strategies for maximizing social media’s benefits while mitigating the harms [5]. However, emerging evidence indicates that these benefits are being inequitably distributed, creating a “digital divide” of unequal access to and use of information and communication technologies [7].

To address this new health challenge, we summarize the central role of social media for public health, discuss the emerging health benefits of digital access, define the magnitude of the digital divide, and present strategies for reducing potentially preventable health inequities arising from the existing measurable digital inequalities [8].

## 2. Social Media as a Tool for Public Health

Social media serves as a tool for health officials, public health agencies, and healthcare professionals to communicate health information [9]. This can be especially useful in times of health crisis, such as pandemics, as social media allows information to be shared quickly and widely and can be updated as crises unfold and evolve. For example, one study from the COVID-19 pandemic in Africa found that the number of social media accounts an individual owned significantly predicted their awareness of prevention strategies [10]. Thus, social media offers the potential to deliver crucial truths ahead of counterproductive falsehoods [11].

Social media has become a leading source of health information—and misinformation—for many individuals [12]. A Pew Research study found around half of Americans received a lot or some information about COVID-19 vaccines from social media [13]. A Saudi Arabian study found that 85% of participants sought health information on social media [14]. Another study from Saudi Arabia showed that 76% of participants used social media to acquire health information [15]. Access to social media offers a pathway to accessing beneficial health information, such as warnings, emergency updates, public health advisories, and valuable resources. Exposure to such information can improve health outcomes.

## 3. Health Benefits of Digital Access and Use

Given the benefits of social media for public health, the digital divide has been found to contribute to disparate health outcomes. This apparent influence was seen during the COVID-19 pandemic in the U.S., as counties with the highest percentages of people without internet access had the highest rates of infection and death but the lowest rates of vaccination [16]. This finding was echoed by a study that showed that a lack of internet access was consistently associated with higher COVID-19 mortality rates [17].

Emerging data support the notion that increased broadband access is associated with a measurable improvement in health behaviors and, in some cases, health outcomes. For example, the prevalence of diabetes drops 9.6 percent with each higher quintile of broadband access in U.S. counties [18]. One longitudinal study on Medicare beneficiaries found that increased broadband access over the study period accounted for 16% of the improvements in outcomes [19]. Other studies have found that internet access correlates strongly with health outcomes, even when controlling for education, income, and rurality [20]. Evidence from Bangladesh indicated that social media users were three times more likely than non-users to follow established health guidelines during the pandemic [21]. Other studies on COVID-19 prevention suggest that social media and internet usage led to improved health outcomes [22]. Weekly internet usage has also been linked to greater knowledge of fertility cycles among females and increased antenatal care in Nigeria and Rwanda, as did ownership of a mobile phone, which also has been associated with greater use of iron supplementation during pregnancy in these countries and Senegal [23]. The inability to access or use social media—a consequence of the digital divide—can be a marker for health outcomes.

We can consider social media access—and digital access at large—to be a social determinant of health. Suppose social media has emerged as a significant source of health information, and yet many people do not have access to or cannot make use of such information. In that case, it seems to follow that such individuals will suffer worse health, as was apparent during the COVID-19 pandemic. Social media is a “non-medical factor that influences health outcomes” [24], thus fitting the WHO’s definition of a social determinant of health.

## 4. Access Problems: Defining the Digital Divide

While social media and digital access continue to increase, around one-third of the world’s population still lacks access to the internet. Lack of access is concentrated in developing nations, even though the fastest growth is occurring in these areas. In Africa, just 40% of the population has digital access, while 64% of Asian and Pacific individuals can access online.

Social media usage has increased in every region of the world over the last five years, yet large regional differences in access remain (Table 1). The greatest increases in social media were seen in Western and Central Asia, while the smallest were in Eastern and Central Africa. Nonetheless, the gap between the highest and lowest narrowed. In 2019, Eastern Asia had the highest usage (74.9%) and Central Africa the lowest (6.0%)—a 12-fold difference. In 2024, Northern Europe had the highest usage (80.2%) and Central Africa again the lowest (9.9%)—an 8-fold difference.

Access disparities exist not only by region but according to gender, age, rural–urban location, and household income. In the 45 least-developed countries, based on the percentage of individuals using the internet in 2022, digital access gaps exist for the female population (30 percent online versus 43 percent of males), the older population (26 percent online among people 25 and older versus 48 percent for 15- to 24-year olds), and rural populations (28 percent online versus 52 percent in urban areas) [27].

While industrialized nations have robust access levels, access remains stratified in these nations. Almost a quarter of American adults from households with incomes below USD 30,000 per year do not own a smartphone; 41% do not own a laptop, and 43% lack home broadband services. Conversely, almost two-thirds of adults in households making over USD 100,000 per year possess more than one device to access the internet, and 93% of such individuals have broadband services [28]. Almost a fifth of people living on tribal lands in the U.S. lack access to broadband; this is true for just 4% of Americans not living on tribal lands [29].

The digital divide is not just about access, however. The ability to use information and communication technologies to find, evaluate, create, and communicate information is defined as “digital literacy” [30]. It also plays a significant role in determining who can access health information through social media and the internet. Previous research has indicated that individuals from disadvantaged groups in the U.S. lack basic navigation skills on digital platforms and instead use the internet primarily for entertainment compared to their wealthier counterparts [7]. Digital literacy is a critical issue in low-income countries: a study from Ethiopia of 193 healthcare providers working in public health centers concluded that basic digital competence was low, especially in problem-solving, safety, and communication [31].

## 5. Actions Needed: Reducing Health Inequities by Reducing Digital Inequalities

Based on social media’s increasing importance in public health influence on health equity, tackling digital inequity should be a priority in public health and global health agendas. Reducing the digital divide is no easy task, but it might be aided by first acknowledging that access to social media and other digital platforms is a social determinant of health.

### 5.1. Enabling Government Policy and Regulation Environment

The Federal Communications Commission of the U.S. has taken a step toward establishing digital access as a social determinant of health through its “Advancing Broadband Connectivity as a Social Determinant of Health Initiative.” Recognizing the positive effects digital access can have on health should be formally considered by individual healthcare professionals and authoritative bodies [18]. Broader digital access could lead to more effective and holistic care to improve health outcomes. This designation might also kindle policies that could creatively incorporate social media and other digital platforms into treatment protocols.

Creating more favorable environments for digital platforms is especially important in countries that intentionally underinvest in connectivity and restrict social media usage. Censorship has continued to spread alongside social media’s expansion [32], potentially preventing individuals from accessing the crucial health-related information available on social media and other digital mediums. For example, the Thai, Indonesian, and Uzbek governments punished individuals for posting information that raised concerns regarding the extent of and proper response to COVID-19, thus preventing individuals from seeing information that raised alarms about the virus [33]. Updated laws and policies within these countries that encourage usage of the internet and social media will allow users to feel more secure in accessing technology and will ultimately lead to more adoption of such technology.

### 5.2. New and Updated Infrastructure to Expand Digital Access for Underserved Populations

Decreasing the access divide will require new infrastructure and updates to existing infrastructure. Public funding is already being allocated to accomplish this goal in some parts of the world. The EU, for example, has allocated 1.5 billion euros in the past few years for the digitalization of countries in Central Europe, which have lagged states in Western Europe. Bulgaria has directed much of these funds to build proper infrastructure for the digital age, while Romania has used the funds to further technology adoption by Romanians [34]. In India, a national digital infrastructure program has significantly reduced poverty through robust investments in digital infrastructure, including broadband connectivity [35].

In the U.S., a landmark infrastructure bill signed into law in 2021 directed USD 65 billion toward ensuring all Americans have access to reliable, affordable, high-speed internet [36]. The law creates a permanent program to help low-income households access online.

Meanwhile, an analysis by the World Bank shows that Africa needs USD 100 billion in critical infrastructure investment, including 250,000 4G stations and 250,000 km of fiber cables, to bring adequate, universal internet to all of Africa [37].

Several private initiatives have also emerged. SpaceX, for instance, has launched a Starlink satellite network that has begun providing internet to 80 countries. Starlink’s competitors and similar companies include Eutelsat, Bigblu Broadband, Digiweb, Maxis Communications, and Constellation networks. These companies are preparing to operate satellite internet constellation systems that will play a key role in reducing the cost of internet connectivity to rural and poor communities. Google has created programs to generate more equitable access through its Next Billion Users Initiative. Meta has invested in improving internet services and fiber infrastructure in Africa, the Middle East, and East Asia. While such companies’ investments are driven by an interest in expanding their consumer base, private actors can help bring more users to social media and other digital platforms, allowing them to interact with useful health information.

In some areas, connectivity will also rely on innovations in electric supply. Many developing countries, especially in sub-Saharan Africa, have limited access to electricity, especially in rural areas [38]. Recent technological developments—primarily among Chinese manufacturers—have dramatically dropped the cost of solar energy. This cost shift has given countries in the Southern Hemisphere a window of opportunity to narrow the digital divide with reliable electricity to power digital infrastructure and gadgets, even in remote and rural locations.

Finally, to encourage and strengthen the use of digital technologies in the Global South, there is a need to invest in establishing and proliferating innovation hubs. These innovation hubs will focus on implementing local programs to counter the imposition of digital solutions from the more developed countries in the Northern Hemisphere. These hubs will foster cooperation, providing more opportunities to advance locally developed solutions.

### 5.3. Making Digital Access and Use Affordable

The digital divide is driven not just by availability but also by affordability. Nearly a third of Americans attribute their lack of broadband to high costs [29]. While these households often have the necessary infrastructure, they cannot afford to pay for broadband services. One U.S. report stated that the affordability gap is the biggest driver of the digital divide in 43 American states [39].

Affordability is a notable barrier globally, too. The cheapest internet-enabled handset devices cost an average of 19% of the monthly income in low- or middle-income countries [40].

Several initiatives are in place to make digital access more affordable, at least for some parts of the world. For example, so-called lightweight operating systems—stripped-down smartphones that are still functional but far less expensive to manufacture and operate—significantly lower the cost of internet usage. Increasing offerings of second-hand and refurbished devices also increases the likelihood of low-cost access. Flexible payment models are useful among low- or middle-income populations, where there are more likely to be unpredictable variations in income [40]. Service-providing companies can also implement alternative payment models for such individuals. For example, research from the Brookings Institute indicates that waiving data caps for people who cannot pay for more costly data plans helps to reduce the affordability gap [41].

### 5.4. Digital Literacy

Given that the digital divide is not just about access but also about usage competency, digital proficiency initiatives are also needed. Such measures are emerging globally. American programs, for instance, have granted USD 60 million to states seeking to improve their digital literacy programs [42]. Such programs are being targeted to young audiences in schools and adult professionals in work environments, offering early exposure to and retroactive awareness of digital navigation skills. Initiatives like these are also popping up in East Asia, where UNICEF has launched programs to bolster girls’ digital literacy [43]. Performance indicators have been proposed for building digital literacy for sustainable development, and their relevance has been illustrated with promising examples of digital literacy programs for rural populations in India as well as low-literacy populations in Kenya, Senegal, Mali, Burkina Faso, and Tanzania [44].

## 6. Conclusions

Educational attainment, economic opportunity, nutritious diets, and secure housing are social determinants that promote better health and longer lives. Now, digital equity should be added to this list. The current digital divide is a systemic issue that exists not only between countries but also within them. Given the large disparities in digital access and proficiency, concerted and sustained action is required.

In our view, the potential health impacts of closing the digital divide will require actions in four critical areas. First, digital equity should be supported by enabling government policy, a more favorable regulatory environment, and inclusion in health policy and healthcare decisions. Second, public and private infrastructure investments are needed to expand the necessary technology in areas that otherwise lack resources. Third, initiatives like stripped-down smartphones and alternate payment models will help improve affordability for resource-poor populations, whose health needs are often the most neglected. And finally, initiatives that help people use digital information more effectively should help to reduce the misuse of social media and vulnerability to dangerous misinformation.

The overall immediate benefit of reducing digital inequities will be an increased access to the internet and social media, which have demonstrated benefits as public health tools that should help to reduce pervasive health disparities.

## Figures and Tables

**Table 1:** Global social media penetration rate * by region, 2019 [25] vs. 2024 [26].

	Social Media Penetration Rate		Change in Penetration Rate 2019 to 2024	Rank Based on Penetration Rate	
	2019	2024		2019	2024
GLOBAL AVERAGE	42.0	62.2	20.2		
EUROPE					
Northern Europe	59.0	80.2	21.2	4	1
Western Europe	45.0	78.2	33.2	10	2
Eastern Europe	40.0	70.0	30.0	12	6
Southern Europe	50.0	73.5	23.5	8	4
ASIA					
Eastern Asia	70.0	74.9	4.9	1	3
Western Asia	46.0	66.9	20.9	9	8
Southeastern Asia	56.0	61.6	5.6	6	10
Central Asia	8.0	37.9	29.9	17	15
Southern Asia	22.0	32.9	10.9	15	16
WESTERN HEMISPHERE					
North America	61.0	70.8	9.8	2	5
South America	61.0	67.7	6.7	3	7
Central America	59.0	66.7	7.7	5	9
Caribbean	44.0	51.3	7.3	11	12
OCEANIA	51.0	59.9	8.9	7	11
AFRICA					
Southern Africa	36.0	42.5	6.5	14	13
Northern Africa	37.0	41.5	4.5	13	14
Western Africa	12.0	16.5	4.5	16	17
Eastern Africa	7.0	10.5	3.5	18	18
Central Africa	6.0	9.9	3.9	19	19

* Social media penetration is social media users as a share of the total population. Users may not represent unique individuals, so figures may exceed internet penetration values.
